# Neonatal mortality and video assessment of resuscitation in four district hospitals in Pemba, Tanzania

**DOI:** 10.1038/s41390-023-02824-7

**Published:** 2023-09-28

**Authors:** Charlotte Carina Holm-Hansen, Stine Lund, Tine Bruhn Skytte, Jil Molenaar, Christina Nadia Steensgaard, Ulfat Amour Mohd, Said Mzee, Said Mouhammed Ali, Jesper Kjærgaard, Gorm Greisen, Jette Led Sorensen, Anja Poulsen

**Affiliations:** 1 Global Health Unit, Department of Paediatrics and Adolescent Medicine, The Juliane Marie Centre, Copenhagen University Hospital, Rigshospitalet, Copenhagen, Denmark; 2 Department of Neonatology, The Juliane Marie Centre for Children, Copenhagen University Hospital Rigshospitalet, København, Denmark; 3 Reproductive and Maternal Health Research Group, Public Health Department, Institute of Tropical Medicine Antwerp, Antwerp, Belgium; 4 Family Medicine and Population Health, Faculty of Medical and Health Sciences, University of Antwerp, Antwerp, Belgium; 5 Public Health Laboratory—Ivo de Carneri, Chake Chake, Pemba, Tanzania; 6 Department of Clinical Medicine, Faculty of Health and Medicine Sciences, University of Copenhagen, Copenhagen, Denmark; 7 The Juliane Marie Centre for Children, Women and Reproduction, Copenhagen University Hospital Rigshospitalet, København, Denmark

## Abstract

**Background:**

We aimed to assess risk factors for neonatal mortality, quality of neonatal resuscitation (NR) on videos and identify potential areas for improvement.

**Methods:**

This prospective cohort study included women in childbirth and their newborns at four district hospitals in Pemba, Tanzania. Videos were analysed for quality-of-care. Questionnaires on quality-of-care indicators were answered by health workers (HW) and women. Risk factors for neonatal mortality were analysed in a binomial logistic regression model.

**Results:**

1440 newborns were enrolled. 34 newborns died within the neonatal period (23.6 per 1000 live births). Ninety neonatal resuscitations were performed, 20 cases on video. Positive pressure ventilation (PPV) was inadequate in 15 cases (75%). Half (10/20) did not have PPV initiated within the first minute, and in one case (5.0%), no PPV was performed. PPV was not sustained in 16/20 (80%) newborns. Of the 20 videos analysed, death occurred in 10 newborns: 8 after resuscitation attempts and two within the first 24 h. Most of HW 49/56 (87.5%) had received training in NR.

**Conclusions:**

Video analysis of NR revealed significant deviations from guidelines despite 87.5% of HW being trained in NR. Videos provided direct evidence of gaps in the quality of care and areas for future education, particularly effective PPV.

**Impact:**

- Neonatal mortality in Pemba is 23.6 per 1000 livebirths, with more than 90% occurring in the first 24 h of life.
- Video assessment of neonatal resuscitation revealed deviations from guidelines and can add to understanding challenges and aid intervention design.
- The present study using video assessment of neonatal resuscitation is the first one performed at secondary-level hospitals where many of the world’s births are conducted.
- Almost 90% of the health workers had received training in neonatal resuscitation, and the paper can aid intervention design by understanding the actual challenges in neonatal resuscitation.

## Introduction

Approximately 2.5 million newborns die annually in the neonatal period, and most of these deaths are preventable by effective interventions delivered across the whole continuum of care during antenatal, intrapartum, childbirth and post-natal care.^1,2^ Additionally, 2.6 million stillbirths occur annually, half of these intrapartum.^3–6^ Most of the mortality occurs in low and middle-income countries (LMIC).^7,8,4^ The 2030 Sustainable Development Goals (SDG) 3.2 sets a target of less than 12 neonatal deaths per 1000 live births.^9^ However, current global projections are not on track to attain the 2030 SDG target.^7,10,11^

Neonatal mortality is greatest in the first 24 h of life, during which approximately 36% of mortalities occur.^3,5^ An estimated 73% of neonatal deaths occur within the first week of life.^3,5^ The leading causes of neonatal death are intrapartum-related events (previously birth asphyxia), infections, and preterm birth complications.^3,7,12,13^ What happens immediately after birth can affect an entire life course.^14,15^ Positive pressure ventilation (PPV) is the key component of neonatal resuscitation, as 5-10% of newborns fail to initiate and sustain adequate breathing at birth.^16–21^ Neonatal resuscitation programmes (NRP) such as Helping Babies Breathe (HBB) can reduce intrapartum-related stillbirths and early neonatal mortality but focus on the whole continuum of reproductive, maternal, and newborn care is needed to increase overall neonatal survival.^15,22–24^

The quality of NR in LMIC and translation of knowledge into clinical practice remains a challenge.^14,15,21^ Video recording has been used to evaluate health workers (HW) neonatal resuscitation (NR) performance and adherence to guidelines, primarily in high-resource settings, with a few recent studies from tertiary hospitals in LMIC.^21,25–33^ Our feasibility study supported that video can be used to understand gaps in quality of care in NR in this context.^34^

This study aimed to assess risk factors for neonatal mortality in the cohort and assess the quality of neonatal resuscitation in four secondary health facilities in Pemba, Zanzibar, through video recordings and identify potential areas for improvement.

## Methods

### Study design

The study was a prospective cohort study, and part of a pre-post intervention trial, the Newborn Emergency Outcome Study, with the aim of reducing neonatal mortality (clinicaltrial.gov NCT040937778, Zanzibar Health Research Institute: NO.ZAHREC/2 August 2019/30). The study was conducted at four district hospitals in Pemba, Tanzania, for 11 weeks from September to November 2019. The STROBE (Strengthening the Reporting of Observational Studies in Epidemiology) checklist was applied for the reporting of the current study.

### Setting

Pemba is an island in the Zanzibar archipelago with approximately 450,000 inhabitants.^35,36^ There are four district hospitals assisting a total of 16,500 births each year.^36^ The facility rate for births is increasing, with 67.6% of all births taking place in health facilities in 2019.^36^ Human resources are scarce, and only a few medical doctors or clinical officers are available at each hospital. The nurse/midwife-to-labouring women ratio is between 1:2 and 1:4, depending on the time of the day. All hospitals have access to instrumental assisted birth and caesarean section. The NR guidelines available in facilities are an HBB poster and guidelines provided by the Ministry of Health and WHO.^19^ The women and their newborn enrolled in this study received standard care, no checklists were used before the childbirth, and partographs were used inconsistently. The available equipment for the management of the newborn consists of a resuscitation table in three out of four hospitals (one hospital used a wooden table with no radiant heater for resuscitation), gloves, bulb suction, a self-inflating bag and mask, and an oxygen source (not always available). No other equipment or physiological data were available or used in this setting (e.g., ECG, oxygen saturation, heart rate measurement or bubble CPAP). Traditional clothes, called a kanga, brought by the women in labour are available for wrapping, drying, and preventing heat loss of the newborn.

### Participants

Women in labour and their newborns, both singleton and multiple gestations were eligible for participation. All stillbirths were excluded and reported in a separate paper.^37^ Participants were enrolled once their hospital admission was completed, and they consented to video recording of the newborn immediate care and evaluation. Consent was confirmed post-partum. If prospective consent was not possible, participants were enrolled after giving consent, as soon as possible after childbirth. All HW at the four maternity wards consented to participate, and no economic incentives were provided.

### Data sources, data collection and management

A motion-triggered camera was used and recorded all instances of newborns being placed on the resuscitation table. We tested the acceptability of video recordings of NR in a feasibility study.^34^ The camera was set at an angle to capture the newborn and the hands of the health worker but shielded so that only the resuscitation bay was recorded, guaranteeing the anonymity of the women and health workers. The camera recorded both video and audio, the latter used solely to determine if the newborn was crying and grunting; no dialogue was analysed since it was beyond the scope of our studies and not included in our original research protocol.

We recruited 18 research assistants with healthcare backgrounds. A postnatal questionnaire was performed and collected sociodemographic, obstetric, birth and neonatal data. Data was stored into RedCap (v5.12.1). The HW in charge of the specific birth filled out the sections specific to obstetric risk factors and the birth outcome based on WHO and national guidelines.^19,18^ Efforts were made to follow up with all study participants by phone when the newborn turned 28 days. A village follow-up was planned but had to be cancelled in March 2020 due to the Covid-19 pandemic.

### Outcomes and variables

Resuscitation of newborns with PPV captured on video were included in the analysis. We used a previously published analytical framework of quality-of-care indicators where resuscitation procedures are scored according to guidelines.^19,34^ The clinical appearance of the newborn was logged as no respiration = 0, gasping = 1 or breathing = 2. The clinical actions performed by the HW were registered in a thematic template, including a description of performance on heat loss prevention, positioning of the newborn’s head, clearing the airway via suction, stimulation, bag and mask ventilation, heart rate assessment and oxygen management. Each intervention was assessed at three levels: properly performed procedures, inadequate performed procedures (delayed intervention or inadequate technique for a given procedure), and procedures omitted or performed but not indicated according to NR guidelines.^19,34^

### Data analysis

All video recordings were analysed by CH. Two independent researchers (CS and SL) randomly selected one-quarter of the videos for quality assessment. The videos were reviewed after the study period, and an individual timeline was produced to document procedures performed in each neonatal resuscitation.

Video observations were registered in Excel (version 16.60, Microsoft Corporation, Washington, United States), and quantitative variables were registered in RedCap. We categorised continuous variables according to common medical standards and newborn risk factors. In descriptive statistics, we used numbers and percentages or median and interquartile range (IQR).

Analyses of differences in continuous variables were performed using independent-sample t-tests. Chi-square tests were used to compare variables. To investigate variables associated with pregnancy and birth outcomes, a binominal logistic regression model was used. Analysis for each confounder was adjusted for the mother’s age and presented with adjusted odds ratios [AOR], including 95% CI. Birth weight was included in the logistic models for the newborn. Twin births were considered two distinct cases in the logistic regression models. Due to the limited number of twin births (*n* = 52), the bias for these samples was considered limited.

All tests were two-sided, and all analyses were considered significant if *p* < 0.05. Estimates were shown with 95% confidence intervals (CI). SPSS (version 28.0, IBM, New York, United States) was used for analysis.

## Results

**Fig. 1:**
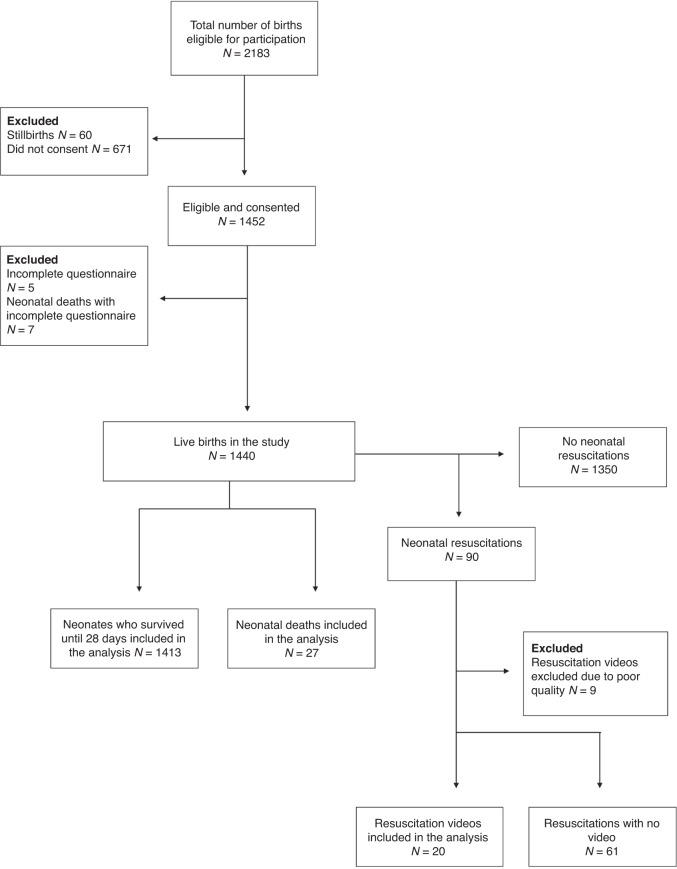
Flowchart of the study population. Inclusion of births in the cohort, stillbirths excluded. The neonatal mortality rate was 23.6 per 1000 livebirths in the cohort.

During the study period, 2183 newborns and their mothers were eligible for participation, and informed consent was obtained for 1440 liveborn newborns. Of 90 neonatal resuscitations, 20 were recorded with sufficient quality to be included in the analysis (Fig. 1). Sociodemographic, education, training, and experience of the 56 participating health workers are presented in Table 1.

**Table 1:** Sociodemographic, education, training, and experience of the participating health workers

	*N* = 56
Sex	
Female	47/56 (83.9%)
Male	7/56 (12.5%)
Missing data	2/56 (3.6%)
Age	
Mean	30.65 ( + −8.3)
Range	21–56 years
Education	
Nurse	28/56 (50%)
Midwife	11/56 (19.6%)
Medical Doctor	7/56 (12.5%)
Clinical officer	3/56 (5.4%)
Assistant nurse	1/56 (1.8%)
Other (health orderly, nursing officer)	6/56 (10.7%)
Training experience	
Helping Babies Breathe	27/56 (48.2%)
WHO Essential Care for Every Newborn	14/56 (25.0%)
WHO Essential Newborn Care	9/56 (16%)
PartoMa Seminar (Including NR training)	34/56 (61%)
At least one training where NR was taught	49/56 (87.5%)
Births conducted in the last month	
<10	20/56 (35.7%)
11–20	23/56 (41.1%)
>21	11/56 (19.6%)
Missing data	2/56 (3.6%)
Neonatal resuscitations performed in the past month	
<1 neonatal resuscitations	11/56 (19.6%)
1–5Neonatal resuscitations	38/56 (67.8%)
>5 neonatal resuscitations	5/56 (9.0%)
Missing data	2/56 (3.6%)
Experience with the care of newborns after resuscitation in the past month	
<1	23/56 (41.1%)
1–5	30/56 (53.6%)
>5	1/56 (1.7%)
Missing data	2/56 (3.6%)
Experience with care of newborns with infection in the past month	
<1	29/56 (51.7%)
1–5	23/56 (41.1%)
>5	2/56 (3.6%)
Missing data	2/56 (3.6%)
Experience with care of a newborn with low birth weight (<2500 gram)	
<1	22/56 (39.3%)
1–5	33/56 (58.9%)
>5	0/56
Missing data	1/56 (1.8%)

### Neonatal mortality, characteristics, and risk factors

During the study period, 34 newborns died in the neonatal period, corresponding to a neonatal mortality rate (NMR) of 23.6 per 1000 live births. Thirty-two newborns died within the first 24 h after birth, one died between day 1 and day 7 and one died between day 7 and day 28. For 7 of newborns who died within the first 24 h, no additional data were available and they are not included in the final analysis of risk factors for neonatal death. Ten of the deaths were recorded, and included in the video analysis; it means half of the resuscitated newborns in the video cohort died. Of the 27 neonatal deaths included in the final analysis, 15 (57.7%) were attempted resuscitated and 14 (51.9%) received bag and mask ventilation. All risk factors for neonatal death can be seen in Tables 2 and 3.

**Table 2:** Sociodemographic and obstetric characteristics of the study population stratified by neonatal death

	Total live births *N* = 1440			
	Neonatal deathsa *N* = 27	Neonates who survived until 28 days *N* = 1413	*p*-value	AOR^b^ (95% CI)
Maternal age				
<20 years	1 (3.8%)	156 (11.2%)	0.30**	0.38 (0.05–2.94)
20–29 years	14 (53.8%)	837 (60.3%)		Reference group
30–39 years	10 (38.5%)	334 (24.1%)		(0.79–41)
>40 years	1 (3.8%)	61 (4.4%)		0.98 (0.13-7-58)
Mean (SD)	28.58 (±7.50)	26.46 (±6.34)	0.047*	1.05^a^ (0.99–1.11)
Missing data	1	25		
Hospital				
Hospital 1	12 (46.2.0%)	523 (37.3%)	0.002**	Reference group
Hospital 2	5 (19.2%)	248 (17.7)		0.99 (0.34–2.88)
Hospital 3	5 (19.2%)	324 (23.1%)		0.74 (0.25–2.14)
Hospital 4	4 (15.4%)	307 (21.9%)		0.62 (0.20–1.97)
Missing data	1	11		
Education				
No formal education	3 (11.5%)	123 (8.8%)	0.66**	1.06 (0.17–6.52)
Primary	8 (30.8.0%)	326 (23.3%)		1.19 (0.25–5.74)
Secondary	13 (50.0%)	870 (62.1%)		0.75 (0.16–3.45)
College or above	2 (7.7%)	83 (5.9%)		Reference group
Missing data	1	11		
Marital status				
Married	25 (96.2%)	1385 (98.5%)	0.33**	Reference group
Unmarried	1 (3.8%)	21 (1.5%)		2.69 (0.35–20.89)
Missing	1	7		
Parity on admission				
Primiparous (1)	6 (23.1%)	426 (30.5%)	0.63**	0.96 (0.33–2.77)
Multiparous (2–4)	11(42.3%)	587 (42.0%)		Reference group
Grandparous (>5)	9 (34.6%)	384 (27.5%)		0.77 (0.26–2.33)
Missing data	1	16		
Gestation				
Singleton	22 (88.0%)	1364 (96.5%)	0.024**	Reference group
Multiple	3 (12.0%)	49 (3.5%)		1.61 (0.44–5.92)
Missing data	2	0		
Previous death of a newborn				
Yes	8 (30.8%)	157 (11.2%)	0.002**	**3.1 (1.26–7.61)**
No	18 (69.2%)	1234 (88.7%)		Reference group
Missing data	1	22		
Previous caesarean section				
Yes	5 (19.2%)	123 (8.8%)	0.07**	2.3 (0.85–6.25)
No	21 (80.8%)	1270 (91.2%)		Reference group
Missing data	1	20		
Attended Antenatal Care (ANC)				
0 times	1 (3.8%)	3 (0.2%)	<0.001**	53.55(4.28–670.57)
1–3 times	17 (65.4%)	715 (51.4%)		1.91 (0.82–4.47)
4 as recommended by the WHO	8 (30.8%)	673 (48.4%)		Reference group
Missing data	1	22		
Decreased fetal movement				
Yes	5 (20.0%)	175 (12.7%)	0.28**	1.74 (0.65–4.71)
No	20 (80.0%)	1204 (87.3%)		Reference group
Missing data	2	34		
No fetal movement				
Yes	5 (20.0%)	19 (1.4%)	<0.001**	19.59 (6.51–58.94)
No	20 (80.0%)	1348 (98.6%)		Reference group
Missing data	2	46		
Manual rupture of membranes				
Yes	9 (34.6%)	760 (55.2%)	0.036**	0.46 (0.20–1.05)
No	17 (65.4%)	616 (44.8%)		Reference group
Missing data	1	12		
Preterm and/or Premature Rupture of Membranes (PROM and/or PPROM)				
Yes	5 (18.5%)	147 (10.5%)	0.18**	2.09 (0.78–5.65)
No	22 (81.5%)	1255 (89.5%)		Reference group
Missing data		11		
Foul smelling amniotic fluid				
Yes	2 (7.4%)	111 (7.9%)	0.96**	1.01 (0.24–4.34)
No	25 (92.6%)	1290 (92.1%)		Reference group
Missing data	1	12		
Amniotic fluid				
Clear	25 (92.6%)	1346 (96.6%)	0.26**	2.34 (0.54–10.21)
Meconium	2 (7.4%)	47 (3.4%)		Reference group
Missing data		20		
Antepartum haemorrhage				
Yes	3 (11.5%)	131 (9.4%)	0.71**	1.34 (0.4–4.55)
No	23 (88.5%)	1268 (90.6%)		Reference group
Missing data	1	14		
Birth mode				
Spontaneous vaginal delivery	21 (77.8.0%)	1297 (92.3%)	0.02**	Reference group
Assisted vaginal delivery	0 (0%)	1 (0.1%)		
Caesarean section	6 (22.2%)	107 (7.6%)		**3.51 (1.38–8.94)**
Missing data		8		
Presentation				
Cephalic	24 (88.9%)	1355 (97.3%)	0.005**	Reference group
Breech	3 (11.1.0%)	28 (2.0)		**5.57 (1.56–19.89)**
Malpresentation	0	9 (0.6.%)		
Missing data		21		

Statistically significant *p*-values are in bold.

^a^The WHO definition for a neonatal death was used: “a death among live births during the first 28 completed days of life.

^b^Adjusted Odd Ratio adjusted for age, *independent sample *t*-test; **Chi-square test, not adjusted for age.

**Table 3:** Neonatal mortality by newborn characteristics and resuscitation care performed according to health workers

	Total live births *N* = 1440			
	Neonatal death^a^ mortality *n* = 27	Neonates who survived until 28 days *N* = 1413	*p*-value	AOR^b^ (95% CI)
Sex of the baby				
Female	7 (26.9%)	700 (49.9%)	0.02**	Reference group
Male	19 (73.1%)	702 (50.1%)		3.29 (1.35–8.00)
Missing data	1	11		
Birth weight				
<1500	2 (7.4%)	0	<0.001*	
1500–2000	2 (7.4%)	9 (0.6%)		15.51 (3.14–76.64)
2000–2500	4 (14.8%)	66 (4.7%)		4.23 (1.40–12.79)
<2500	19 (70.4%)	1326 (94.7%)		Reference group
Mean	2.75 (±0.88)	3.21 (±0.51)	<0.001*	0.20 (0.1–0.4)
Missing	0	12		
Did the baby cry immediate after birth				
Yes	13 (48.1%)	1275 (90.7%)	<0.001**	Reference group
No	14 (51.9%)	131 (9.3%)		10.05 (4.53–22.32)
Missing data		7		
Apgar 1				
<4	5 (20.8)	6 (0.5%)	<0.001**	52.82 (11.86–235.16)
4–6	5 (20.8)	55 (4.3%)		5.90 (1.98–17.55)
>7	14 (58.3)	1208 (95.2%)		Reference group
Missing data	3	144		
Apgar 5				
<4	3 (13.6%)	1 (0.1%)	<0.001**	208.37 (14.84–2925.04)
4–6	5 (22.7%)	18 (1.5%)		16.07 (4.82–53.53)
>7	14 (63.6%)	1201 (98.4%)		Reference group
Missing data	5	193		
Apgar 10				
<4	2 (9.5%)	0	<0.001**	
4–6	3 (14.3%)	6 (0.5%)		21.74 (4.10–115.28)
>7	16 (76.2%)	1218 (99.5%)		Reference group
Missing data	6	189		
Resuscitation attempted				
Yes	15 (57.7%)	75 (5.7%)	<0.001**	17.65 (7.54–41.30)
No	11 (42.3%)	1240 (94.3)		Reference group
Missing data	1	98		
Bag and mask ventilation performed				
Yes	14 (51.9%)	66 (4.7%)	<0.001**	17.70 (7.73–40.49)
No	13 (48.1%)	1326 (95.3%)		Reference group
Missing data		21		
Suction performed				
Yes	18 (66.7%)	295 (79.0%)	<0.001**	6.45 (2.82–14.72)
No	9 (33.3%)	1107 (21.0 %)		Reference group
Missing data		11		
Antibiotics administered to the newborn immediately after birth				
Yes	10 (37.0%)	26 (1.9%)	<0.001**	18.19 (7.42–44.99)
No	17 (63.0%)	1363 (98.1%)		Reference group
Missing data		24		

^a^The WHO definition for a neonatal death was used: “a death among live births during the first 28 completed days of life.

^b^Adjusted for birthweight continuous variable.

**Chi-square test, *Independent sample *t*-test.

Significant risk factors for neonatal death were older maternal age, multiple gestations, previous death of a newborn, no antenatal care attendance (ANC), no foetal movement, manual rupture of membranes, breech presentation, caesarean section, male sex, low birth weight, no immediate cry after birth, APGAR score <7 at 1, 5 and 10 min, resuscitation attempt, PPV, suction, and administration of antibiotics to the newborn immediately after birth (Tables 2 and 3).

### Resuscitation videos and adherence to guidelines

**Fig. 2:**
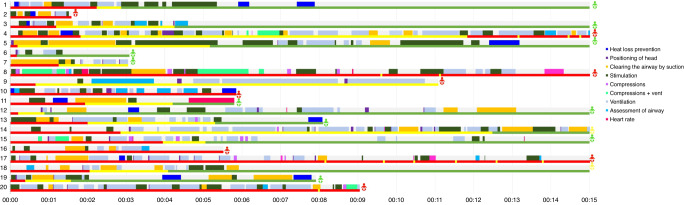
Timeline of interventions in 20 newborns who were resuscitated. The first bar per case is interventions performed on the newborn. Timeline of interventions in 20 newborns who were resuscitated. The first bar per case is interventions. performed on the newborn. The second line per case represents a breathing score where  = 0 (not breathing) , = 1 (gasping) , = 2. (breathing. The infographic at the end of each line represents the neonatal outcome where  = survived to 28 days,  = died. within 24 hours after birth  = immediate neonatal dead.

A total of 90 newborns were resuscitated, of which 20 were judged to be adequately recorded. The reasons for exclusion were: (1) The camera was shielded at the time of the resuscitation due to other patients in the same room who did not provide their consent, (2) Consent was not obtained before birth due to late presentation, labour pain, or an obstetrical emergency, (3) The resuscitation was performed in another location. (4) Technical camera issues. The remaining videos were analysed for quality of care (Table 4), and timelines for each resuscitation were created (Fig. 2).

**Table 4:** Adherence to neonatal resuscitation guidelines assessed by video recording.

Quality of resuscitation assessed by video recordings in 0–15 min of life		
	*n* = 20	Median (IQR) (Range) (Min-max) hh:mm:ss
Heat loss prevention		
Not performed	2 (10%)	
Inadequately performed *(Newborn dried with cloth but cloth not replaced by a new one; head not covered)*	13 (65%)	
Well performed *(Newborn dried and cloth replaced; wrapped and head covered)*	5 (25%)	
Time to first intervention (*n* = 10)		00.02.49 (00.04.48) (00.00.06–00.12.36)
Number of interventions/newborn (*n* = 10)		1 (0) (1–3)
Total time spent on heat loss prevention (*n* = 10)		00.00.20 (00.00.39) (00.00.05–00.00.57)
Positioning of head		
Not performed	2 (10%)	
Inadequately performed *(Head hyperextended or bent to the side)*	4 (20%)	
Well performed *(Head in a sniffing position)*	14 (70%)	
Time to first intervention (*n* = 17)		00.00.58 (00.01.37) (00.00.01–00.05.19)
Number of interventions/newborn (*n* = 17)		2 (3) (1–9)
Total time spent on positioning of the head (*n* = 17)		00.00.04 (00.00.09) (00.00.01–00.01.16)
Clearing the airway via suction		
Not performed when indicated (meconium)	0 (0%)	
Inadequately performed *(Done after the first minute of life; longer than 5* *s; incorrect order (nasal suction before oral); excessive number of times)*	16 (80%)	
Well performed (*or not performed when not indicated)*	4 (20%)	
Time to first intervention (*n* = 17)		00.00.59 (00.01.05) (00.00.01–00.10.03)
Number of interventions/newborn (*n* = 17)		12 (10) (3–35)
Total time spent on suction (*n* = 17)		00.01.05 (00.01.13) (00.00.12–00.02.57)
Stimulation		
Not performed *(Indicated when inactive, apnoeic/not spontaneously breathing or gasping)*	1 (5%)	
Inadequately performed *(Stimulation performed on other places than the back or soles of the feet; too aggressively; excessive number of times)*	8 (40%)	
Well performed	11 (55%)	
Time to first intervention (*n* = 20)		00.00.30 (00.01.12) (00.00.00–00.04.37)
Number of interventions/newborn (*n* = 20)		4.5 (6) (1–15)
Total time spent on stimulation (*n* = 20)		00.00.58 (00.01.36) (00.00.09–00.05.42)
Bag and mask ventilation		
Not performed *(When indicated)*	1 (5%)	
Inadequately performed *(Initiation after the first minute of life; incorrect mask size; incorrect rate (not 40–60* *rpm); incorrect technique (mask turned wrong way); mask leak; not re-evaluated for response after 30* *s; unsustained ventilation*	14 (70%)	
Well performed	5 (25%)	
Time to first intervention (*n* = 19)		(00.01.40) (00.00.10–00.06.56)
Number of interventions/newborn (*n* = 19)		5 (5) (1–20)
Total time spent on bag and mask ventilation (*n* = 19)		00.01.24 (00.01.51) (00.00.17–00.08.01)
Heart rate assessment		
Not performed	12 (60%)	
Inadequately performed *(Performed by feeling the umbilicus)*	7 (35%)	
Well performed *(Performed with stethoscope)*	1 (5%)	
Time to first intervention (*n* = 7)		00.00.07 (00.06.31) (00.01.51–00.13.10)
Number of interventions/newborn (*n* = 7)		1 (2) (1–3)
Total time spent on heart rate assessment (*n* = 7)		00.00.28 (00.01.01) (00.00.10–00.01.14)

The number of interventions refers to the number of (separate) episodes of that intervention.

*s* seconds.

Deviation from resuscitation guidelines on camera adapted and pilot tested in our feasibility study.

PPV initiation was inadequately performed in 15 cases (75%). The mean time to first ventilation was 98 s (10–416 s). Timing was started when the newborn was placed on the resuscitation table. Within the first minute of life, nine patients (45%) were ventilated and 10 (50%) were not ventilated, and in one case (5.0%) no PPV was performed. Suction was performed vigorously and repeatedly in 16 patients (80%), and a median time of 65 s (12–177 s) was spent suctioning. In all cases, ventilation was initiated but not sustained (or not performed at all) with breaks between efforts. On average, in the first 15 min or until resuscitation was ceased, no intervention was performed one-third of the time.

Of the 20 resuscitation recordings, 10 (50%) died; eight after attempting resuscitation (median time spent on resuscitation: 480 s (95–3632 s), and two died in the subsequent hours after initially successful resuscitation.

## Discussion

### Main findings

We identified 34 newborns who died in the neonatal period, corresponding to a neonatal mortality rate (NMR) of 23.6 per 1000 live births. More than 90% of the deaths occurred within the first 24 h of life. To our knowledge, this is the first study to use video recording to assess NR performance in secondary-level district hospitals in a resource-constrained setting. Our study found several deviations in the quality of care on all NR indicators assessed and low adherence to NR guidelines (Table 4). Particularly NR performance for ventilation, which is the most important NR intervention, was suboptimal; it was not initiated within the first minute (or at all) in 55% of the cases, unsustained (or not performed at all) with frequent interruptions in 75% of the cases. Furthermore, other tasks not indicated by NR guidelines were being performed, such as vigorous suction. In one-third of the time, no intervention was performed.

### Interpretation

In our study, 87.5% of HW had attended at least one training in neonatal resuscitation, and 70% of the HW performed resuscitations on a regular basis. Despite this, our findings documented challenges in adherence to NR guidelines, which highlights that NR programmes cannot stand alone. Transfer of competencies learned during training into clinical practice remains a key challenge. A study from Nepal using HBB with a quality improvement cycle showed improved adherence to NR guidelines.^38^ They attribute this success to a multifaceted intervention involving local leadership, multidisciplinary quality improvement teams, daily debriefings, root-cause analysis of poor NR performance and development of inclusive quality improvement goals.^38^ In addition, a systematic review from 2020 of the HBB programme from its initiation in 2010 found a reasonable translation of knowledge and skills.^39^ However, few studies have documented the transfer of knowledge into clinical practice as reflected in neonatal outcomes.^39,40^ A HBB review on the effect on intrapartum-related stillbirths and neonatal mortalities found mixed results on mortality reduction, which further supports that training with frequent refreshers could aid in preserving knowledge and skills.^40^ Thus, there is a need to re-think traditional training and, to a greater extent, support the implementation of learned knowledge and skills into clinical practice.

Videos can help to recognise and monitor essential areas of improvement and aid intervention design. The insights from the videos could not have been obtained by any other means. Direct observations by research assistants could provide some structured observations about NR but cannot provide a real-time recording of NR for analysis and understanding of the actual challenges. Furthermore, direct observations generate a number of ethical issues where an observer should be a trained clinician in order to observe such a complex clinical situation as NR, but a trained clinician should obviously intervene in life-threatening situations. Many studies, both from high-resource settings and a few from low-resource settings, support videos to understand the quality of care.^21,25–33^ Video recordings are beneficial for understanding NR, and our study from Pemba proved that video recordings are also beneficial to understand gaps in the quality of recommended essential newborn care and emphasizes the need for improved post-natal care of healthy newborns to prevent morbidity and mortality.^41,42^ Similarly, a study from Nepal reports that emphasis on post-natal care is paramount to sustain gains in survival after resuscitation and NR programmes.^24^

Our findings stress the need to prioritise effective PPV since oxygenation and reduction of shunts are the key interventions to reverse hypoxia.^16^ Our results are consistent with previous studies from LMIC such as Nepal, Mozambique, and Uganda which found unsustained ventilation and delays in establishing ventilation.^21,27,29,32,33,43^ The 2021 European Resuscitation Guidelines recommend the omission of suction even for newborns born in thick meconium as it delays ventilation and there is an absence of evidence of benefit.^17^ It has been argued that the suction device should be removed from the resuscitation table and observations from our study support this as critical time is diverted to suctioning instead of ventilation.^27^ The AMANHI study attributed perinatal asphyxia as the leading cause of death responsible for more than 47% of neonatal deaths in Pemba.^35^ In addition, the Zanzibari Ministry of Health reports birth asphyxia as the leading cause of death in children under 13 years, accounting for 25.2 % of deaths.^44^

Lastly, we report an NMR of 23.6 per 1000 live births, with more than 90% of the deaths occurring within the first 24 h. Our one-day neonatal mortality is higher than most of the literature, suggesting that the overall neonatal mortality rate in Pemba could be much higher than we report.^3–5,13^ The NMR in our study is slightly higher than the official numbers from the Zanzibari Ministry of Health and the AMANHI-study group.^35,45^ The identified NMR is almost twice as high as the United Nations Sustainable Development Goal specific goal of less than 12 per 1000 livebirths by 2030.^9^

Challenges in provision of quality of care according to guidelines have many reasons beyond the capacity of HWs, including structural barriers such as lack of human resources, lack of equipment and logistical challenges. Maaløe et al. recommend local adaptation of guidelines, so they are achievable and contextualised to the setting.^46^ In addition, there is a need to understand the barriers to adhere to the guidelines, such as HBB and similar NRP, to succeed and translate into improvements in knowledge and skills and improve neonatal outcomes. Novel technology such as mHealth tools are widely available. A study showed that the Safe Delivery App aids knowledge and skill retention with a non-significant reduction of perinatal mortality, and mHealth solutions such as this could be part of the solution.^47^

### Strengths and limitations

A significant strength of this study is the video as a data source, allowing the definition of birth care in the time resolution of seconds and perhaps reducing the Hawthorne effect of an observer being present.^48^ Another strength is the study of four moderate volume district hospitals, as most of the research from LMIC is from high volume tertiary hospitals. This study, however, also has limitations. Our enrolment rate was 66% and the ethical consent procedures did not include a waiver of consent. Seven women who were enrolled before birth and suffered an immediate neonatal death did not want to participate with further information. Another limitation was that our study’s village follow-up component was made impossible by Covid-19, resulting in a nearly 40% loss-to-follow-up to 28 days which most likely missed some neonatal deaths. Finally, 61 resuscitations were not captured on video. Reasons hereof have been listed previously.^34^

## Conclusion

In conclusion, our study found a neonatal mortality rate of 23.6 per 1000 livebirths and more than 90% of these occurred within the first 24 h after birth. Our study found that video recording of neonatal resuscitation in secondary-level district hospitals in a low-resource setting revealed significant derivations from NR guidelines despite nearly 90% of health workers having received training in NR. The video recordings provided direct evidence of gaps in quality of care, and were instrumental in identifying areas for future education, particularly efforts on effective bag- and mask ventilation.

### Supplementary information

Checklist

## Data Availability

The data is available upon reasonable request from the corresponding author.
